# Myocardial blood flow in patients withType 2 Diabetes Mellitus and normal coronary angiography

**DOI:** 10.1186/1532-429X-13-S1-P275

**Published:** 2011-02-02

**Authors:** Abdulghani M Larghat, John Biglands, John P Greenwood, Tim A Fairbairn, Neil Maredia, Aleksandra Radjenovic, Stephen Ball, Sven Plein

**Affiliations:** 1 University of Leeds, Leeds, UK

## Introduction

Type 2 Diabetes Mellitus (T2DM) is associated with an increased risk of heart failure independently of underlying coronary artery disease (CAD) [1]. Myocardial blood flow (MBF) and MBF reserve can be abnormal in patients with T2DM due to myocardial microvascular dysfunction, contributing to the development of heart failure [2]. The myocardial layers may be affected differently by this disease process.

## Objectives

1. To measure MBF and MBF reserve in T2DM patients without CAD and compare them with age-matched non-diabetics.

2. To compare MBF and MBF reserve in separate myocardial layers.

## Methods

Patients without coronary artery stenosis >30% on invasive angiography were recruited and divided into T2DM and Non-Diabetic groups according to their medical history and blood levels of glycolated haemoglobin. Non-Diabetics had a fasting HbA1c of less than 6% and body mass index less than 30 kg/m2. Patients underwent first pass perfusion CMR on a 1.5T Philips Intera system during adenosine induced hyperaemia (140 mcg/kg/min; 0.05 mmol/kg Gd-DTPA) and at rest. A pulse sequence optimised for acquisition of a single midventricular slice at systole was used (saturation recovery segmented gradient echo, 2 x SENSE, TR 2.7ms /TE 1.0ms/flip angle 15°, typical FOV 380x380 mm, matrix 160x160, slice thickness 10 mm). Endocardial and epicardial contours were drawn using MASS (version 6, Leiden, Netherlands) with the slice segmented into transmural, epicardial and endocardial layers. Absolute MBF at stress and rest was calculated using Fermi Function deconvolution [3]. MBF Reserve was calculated as stress values divided by rest values.

## Results

Results are reported as mean (±SD). Sixteen patients with T2DM (12 males, age= 60 ±6 years, BMI = 31 ±4.6) and 16 non-diabetics (6 males, age= 60±9 years, BMI= 26.9±2.4) were studied. Patients with T2DM had significantly higher resting MBF in all myocardial layers (transmural, endocardial and epicardial) (Table 1). Transmural MBF reserve was significantly lower in T2DM patients compared to non-diabetics (Figure; 1A-1C.)

**Table 1:** shows global MBF (ml/g/min) at rest, rest and the reserve means for both groups

	T2DM , n=16	Non-T2DM, n=16	p Values
Rest Transmural	1.70±0.98	1.07±0.27	0.02
Rest Epicardium	1.73±0.89	1.37±0.58	0.06
Rest Endocardium	1.54±0.94	1.05±0.30	0.18
Stress Transmural	3.30±1.20	3.16±1.28	0.75
Stress Epicardium	3.52±1.49	3.18±1.23	0.48
Stress Endocardium	2.87±1.06	2.98±1.08	0.77
Transmural Reserve	2.10±0.74	2.96±1.06	0.02
Epicardial Reserve	2.59±1.07	2.95±1.12	0.36
Endocardial Reserve	1.85±0.67	2.33±0.91	0.09

**Figure 1:**
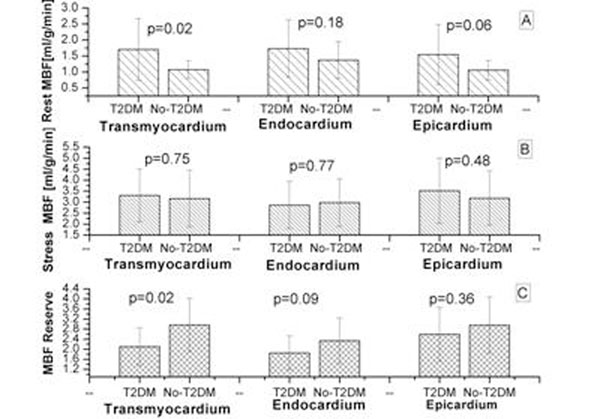
Mean MBF means at rest (1.A), at stress (1.B) and MBF reserve (1.C) in both groups

## Conclusion

Type 2 diabetic patients when compared to age-matched non-diabetics have higher resting transmural MBF but a lower MBF reserve. The endocardial and epicardial layers are equally affected, although in the present study these differences were not statistically significant.
